# Knowledge, Attitude, and Practice About the Process of Genetic Counselling Among Clinicians

**DOI:** 10.7759/cureus.45883

**Published:** 2023-09-24

**Authors:** Jyoti P Kulkarni, Sangeetha Arumugam, Nandha Kumar Subbiah, Joy A Ghoshal

**Affiliations:** 1 Genetics Unit, Department of Anatomy, All India Institute of Medical Sciences, Mangalagiri, Mangalagiri, IND

**Keywords:** genetical counseling, genetic screening, pedigree, barriers to genetic testing, attitude of health personnel

## Abstract

Introduction

Clinicians agree with the fact that the impact of genetics in the field of medicine is humongous. They have to cope with the rapid advances in the field of clinical genetics and offer the best treatment to the patients at the right time. Disease with an underlying genetic cause not only involves the patient but also the family and the community. In the process of genetic counseling, the patient and the family are educated about the genetic basis of the disorder. This helps the patient and the family to make a well-informed decision. It also helps to reduce the genetic burden of the disease in the community over a period of time. In this regard, knowledge, attitude, and practice about the process of genetic counseling among clinicians is imperative.

Methods

A structured pre-validated questionnaire was distributed amongst 60 clinicians from different departments. Their responses were assessed based on the Likert scale. The data obtained were analyzed using descriptive statistics and expressed in percentages.

Results

In the present study, nearly 90% of the clinicians felt that it was important to gather a multi-generational family history of the patient and advise them about inheritance patterns, recurrence risk, and genetic tests for a disorder with an underlying genetic cause. The need to educate the family members regarding the importance of genetic tests and referral to appropriate support groups if they test positive for a genetic disorder receive a positive response. Mostly the participants agreed that parents of children and couples at risk of having a child affected by a genetic disease should undergo genetic counseling.

Conclusion

Clinicians may not always be aware of the underlying genetic cause and genetic tests available or may face a paucity of time to counsel the patient and the family. Genetic counseling needs to be done at length in multiple sessions, and it is essential to reduce the burden of genetic disorders in society.

## Introduction

India is the most populous country in the world [1]. The country has a diverse population of genetic variability with several endogamous groups. Consanguinity, endogamy, and a sharp fall in the infant mortality rate have led to the high prevalence of genetic disorders in the country [2]. Genetic counseling plays a vital role in the early detection of genetic diseases, which will help in both prevention and timely healthcare. The term ‘genetic counseling’ was coined by Sheldon Reed [3] in 1947. It is defined as a communication process that deals with the special health service that provides information and support to the people who have or may be at risk of genetic disorders in a family [4]. It addresses concerns relating to the development and or transmission of a hereditary disorder. In this process, a trained counselor helps the individual and the family to comprehend the medical facts associated with the genetic disorder. This helps the family to deal with the disorder and choose a course of action accordingly. The proband and the family can use the information in a meaningful way, thus minimizing the psychological distress and coping with the disorder. Counseling sessions help decide about the test, adjust to a diagnosis or risk, reduce suffering due to loss, and explore other options [5]. A genetic counselor acts as a bridge between the clinician and the consultant. The concept of genetic counseling has a history of more than 50 years in the United States but still has limited exposure and penetration in India. As of today, genetic counseling has expanded its roots in almost all areas of the medical field. The rapid progress in the field of knowledge and technology has resulted in a huge impact of genomic medicine in many areas of the medical field. The use of genetic testing in clinical practice has increased significantly over the last decade. As genetic testing develops with the growth of new genetic technologies, there is a growing need for genetic counseling [6]. Moreover, evidence-based genetic counseling leads to positive outcomes for people with complex diseases. This makes it essential to add genetic services to the field of medicine [7]. Reports have shown to increase in patient empowerment and awareness following cardiovascular genetic counselling [8]. Genetic counseling leads to increased positive health behaviors, improved risk perception accuracy, and decreased anxiety and decisional conflict [9]. Hence, a medical professional has to keep abreast of the developments in genetics and train himself or herself in this area of counseling. In this background, our study is undertaken to evaluate the knowledge, attitude, and practice of the process of genetic counseling among clinicians.

## Materials and methods

Study design

This cross-sectional study was conducted from July to December 2022 by the Genetics Unit, Department of Anatomy, All India Institute of Medical Sciences, Mangalagiri.

Study setting 

All India Institute of Medical Sciences, Mangalagiri, is a multi-specialty tertiary care teaching hospital funded by the Central Government of India. 

Ethical clearance

Before the commencement of the study, ethical clearance was obtained from the Institutional Ethical Committee (AIIMS/MG/IEC/2022-23/163 dated 08.07.2022).

Data collection tool

A structured pre-validated questionnaire was distributed amongst 60 clinicians through Google Forms. The questionnaire was devised from the existing studies and discussions with experts. It was written in the English language, and it consisted of 15 items pertaining to the knowledge, attitudes, and practices about the process of genetic counseling among clinicians. 

Data analysis

The questionnaire was assessed on a Likert scale of strongly agree, agree, strongly disagree, disagree, and neither agree nor disagree. Filling up the questionnaire was voluntary, and anonymity was maintained. All data from the questionnaire were entered into an Excel spreadsheet. To check for the accuracy of the entered data, 10% of the questionnaires were selected randomly and rechecked. The data, thus, collected were analyzed using descriptive statistics (i.e. percentages).

## Results

**Figure 1:**
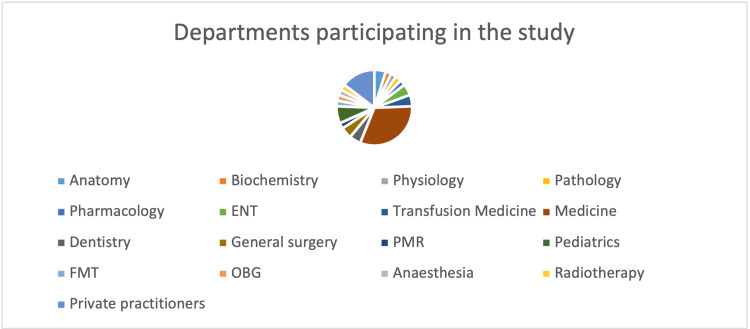
Pie chart showing the department-wise distribution of the clinicians participating in the study

The questionnaire was distributed to all the participants by email. It was answered by 42 out of 60 clinicians from various departments (Figure 1). About 85% of the clinicians who participated in the study were working in the same Institute as that of the authors (All India Institute of Medical Sciences, Mangalagiri) under various departments, such as General Medicine, Pediatrics, OBG, ENT, General Surgery, Anesthesiology, Radiotherapy, etc, and 15% of clinicians were private practitioners. The responses obtained through Google Forms regarding the knowledge assessment, attitude assessment, and practice assessment based on a Likert scale were tabulated and expressed in percentages.

The majority of the participants agreed (19%) or strongly agreed (73.8%) with the fact that gathering a multi-generational family history of the patient is essential to finding if a disease is inherited or not. Similarly, participants agreed (40%) or strongly agreed (47.6%) that it is necessary to refer the patient for appropriate genetic testing to determine the risk and avoid further transmission of the disease. Participants agreed (50%) or strongly agreed (40.5%) to the need to be aware of the genetic disease common in a particular region. When it comes to advising genetic tests, a difference of opinion was observed among the participants, with 23.8% strongly disagreeing, 38.1% disagreeing, 11.9 % neutral, and 23.8% agreeing (Table 1).

**Table 1:** Knowledge of the clinicians regarding genetic counseling

No.	Questionnaire	Response				
		Strongly Disagree	Disagree	Neither Agree/Disagree	Agree	Strongly Agree
1	It is essential to gather genetic family history information, including an appropriate multi-generational family history of a disorder with an underlying genetic cause.	7.1%	-	-	19%	73.8%
2	It is not always necessary to inform the patient to do appropriate genetic tests for risk determination of disease transmission.	23.8%	38.1%	11.9%	23.8%	1%
3	It is necessary to inform the patient to do appropriate genetic tests for risk determination/disease transmission.	7.1%	5%	-	40%	47.6%
4	It is essential to identify/recognize genetic diseases common in a particular region/area.	7.1%	5%	-	54.8%	31%
5	It is important to understand the ethical regulations and techniques of genetic counseling.	7.1%	-	2%	50%	40.5%

Participants agreed (38.1%) or strongly agreed (52.4%) that it is essential to communicate with patients about their genetic condition. Regarding the importance of educating the patient about the aftermath of detecting genetic disorders, participants agreed (47.6%) or strongly agreed (42.9%) with the need to educate the patients and their family members. Participants were of the opinion (agree: 42.9%, or strongly agree: 52.4%) that families should know about the prenatal genetic test and its importance in early detection of genetic conditions. Moreover, they agreed (31%) or strongly agreed (61.9%) that parents of children and couples at risk of having a child affected by a genetic disease should undergo genetic counseling. They disagreed (33%) or strongly disagreed (54.8%) with not providing genetic counseling to affected family members and couples at risk (Table 2).

**Table 2:** Attitude of the clinicians regarding genetic counseling

No.	Questionnaire	Response				
		Strongly Disagree	Disagree	Neither agree or Disagree	Agree	Strongly Agree
1	It is necessary to communicate with the patients regarding their genetic condition and its implications.	7.1%	-	-	38.1%	52.4%
2	It is important to educate the patient about the psychological stress their family members may experience as a result of the detection of a genetic disorder.	7.1%	-	2%	47.6%	42.9%
3	Family should know about the prenatal genetic test and its importance in the early detection of genetic conditions.	5%	-	-	42.9%	52.4%
4	The parents of children and couples at risk of having a child affected by a genetic disease should undergo genetic counseling.	7.1%	-	-	31%	61.9%
5	The parents of children and couples at risk of having a child affected by a genetic disease need not undergo genetic counseling.	54.8%	33%	-	5%	5%

During clinical practice, the need to explain the basic concept of probability of disease, susceptibility, and influence of genetic factors was agreed (57.1%) or strongly agreed (26.2%) by the clinicians. They supported appropriate referral to the support groups by agreeing (38.1%) or strongly agreeing (47.6%). Participants backed carrier testing of family members: agree (31%) or strongly agree (61.9%). The majority of the participants agreed (59.5%) or strongly agreed (31%) with the use of audio-visual aids to perform genetic counseling, which may have more impact on the proband and the family members. There was a mixed response, namely, strongly disagree (7.1%), disagree (9.5%), neutral (11.9%), agree (52.4%), and strongly agree (19%), for informing the patient to do appropriate genetic tests for risk determination of disease transmission (Table 3).

**Table 3:** Practice of the clinicians regarding genetic counseling

No.	Questionnaire	Response				
		Strongly Disagree	Disagree	Neither agree or Disagree	Agree	Strongly Agree
1	It is always essential to explain the basic concept about the probability of disease, susceptibility, and influence of genetic factors on the maintenance of health and development of disease.	5%	5%	10%	57.1%	26.2%
2	A patient with a genetic disorder should be referred to an appropriate support group.	7%	-	9.5%	38.1%	47.6%
3	It is necessary to tell the patients about the potential limitations of the genetic information to be conveyed to other family members.	7.1%	9.5%	11.9%	52.4%	19%
4	A person diagnosed with a genetic disorder should be informed about carrier testing of family members if required.	2%	-	-	50%	47.6%
5	The use of information technologies (Charts/videos) to perform genetic counseling may have more impact on the proband and the family.	1%	-	9%	59.5%	31%

## Discussion

As genetic testing expands with the growth of new genetic technologies, there is an emerging need for genetic counseling, which has become increasingly integrated into all areas of healthcare [10]. It is likely that clinicians will increasingly be asked to communicate and manage the results of genetic testing [11]. In this scenario, we assessed the status of knowledge, attitude, and practice about the process of genetic counseling among clinicians using a structured pre-validated questionnaire. A total of 42 clinicians from various broad specialties participated in the present study by responding to the questionnaire. About 19% of participants agreed and 73.8% strongly agreed to the fact that gathering a multi-generational family history of the patient is necessary to find if a disease is inherited or not. However, 7.1% of the clinicians disagree about the fact of eliciting a genetic family history of a patient and communicating it to the patients and their families. According to Loukas et al. [12], Sushrut Samhita depicts the fact that ancient clinicians and surgeons in India were aware of various congenital diseases and their treatment based on observational studies conducted on aborted fetuses. In the present era too, clinicians are well versed with the fact that most of the diseases have an underlying genetic cause, and the patients along with their families should be made aware of it. About 40% of participants agreed and 47.6% of participants strongly agreed that it is necessary to refer the patient to undergo genetic tests to determine the risk and avoid further transmission of the disease. However, around 12% of participants were of the opinion that it is not always essential to inform the patient about genetic disorders and advice on appropriate genetic tests for a genetic disorder. In a study done by Venugopal et al. [13] on genetic counseling by psychiatrists, 24% of psychiatrists reported the non-feasibility of genetic counseling in day-to-day practice. This is probably due to a lack of awareness about the genetic basis of the disorder and a lack of awareness about the appropriate genetic tests available. Low awareness of the importance of genetic testing leads to an increase in the incidence of hereditary disorders [14]. About 54.8% of participants agreed and 31% of participants strongly agreed with the need to be aware about the genetic disease common in a particular region. When it comes to advising genetic tests, difference of opinion was observed among the participants with 23.8% strongly disagreeing, 38.1% disagreeing, 11.9% neutral, and 23.8% agreeing. Eyes do not see what the mind does not know. Sometimes the clinicians are not well versed in the genetic etiology and different kinds of genetic tests that are available. About 50% of the participants agreed and 40.5% of the participants strongly agreed to the fact that genetic counseling should be done with proper ethical regulations. The findings of the present study concur with the study done by Wonkam et al. [15] who reported that the physicians have an acceptable level of knowledge of clinical genetics, although the awareness of DNA diagnosis seemed poor. In a study done by Diamonstein et al. [16], 75% of physicians reported that they had noticed an increased impact of genetics in their field, while 20% reported that genetics was an integral part of their specialty. A study conducted among practicing physicians and medical students showed insufficient knowledge of the etiology, epidemiology, and prevalence of rare diseases, and many had problems with separating rare diseases from more common disorders [17]. Rare genetic diseases are becoming a public health concern in India. Genetic testing approaches have been demonstrated to accelerate the diagnosis of rare genetic diseases and reduce the socio-economic burden [18]. Knowledge about the genetic contribution to several medical conditions is increasing and will feature across health services impacting most healthcare professionals in the near future [19].

In the current study, participants agreed (38.1%) or strongly agreed (52.4%) that it is essential to communicate with patients about their genetic condition and 47.6% participants agreed or 42.9% participants strongly agreed to the fact that it is important to educate the patients and the family members about the psychological stress the family may experience as a result of detection of a genetic disorder. This fact is not only imperative in the management of the disease condition but also to prevent and curtail the stigmatization of the proband and the family. Informing families about the genetic condition and associated risks may enable them to undergo early screening, prevention, and treatment or to utilize reproductive technologies to avoid having an affected child [20]. Participants were of the opinion, namely, agree (42.9%) or strongly agree (52.4%), that families should know about the availability of prenatal genetic tests and their importance in early detection of genetic conditions. Moreover, they agreed (31%) or strongly agreed (61.9%) that parents of children and couples at risk of having a child affected by a genetic disease should undergo genetic counseling. Given the high burden of the disease and carrier rate in India, screening to identify carriers of genetic disorders with subsequent counseling and prenatal testing for at-risk families is essential to decrease the disease burden [21]. The availability of prenatal diagnostic tests for a number of genetic disorders makes counseling and decision-making much easier. Participants disagreed (33%) or strongly disagreed (54.8%) with the fact that affected parents of children and couples at risk need not undergo genetic counseling. When it comes to attitude toward practicing genetic counseling, practicing physician’s specialty and experience will influence the attitude.

With our findings, the need to explain the basic concept of the probability of disease, susceptibility, and influence of genetic factors was agreed (57.1%) or strongly agreed (26.2%) by the clinicians. They supported appropriate referral to the support groups by agreeing (38.1%) or strongly agreeing (47.6%). In a study done by Truong et al. [22] on primary care physicians, it is reported that 48% of primary care physicians would offer genetic testing, while 71% of primary care physicians would offer referrals to the patients. In a study done by Westwood et al. [23], the appropriate overall referral rate was higher among general practitioners. Participants supported carrier testing of family members with the following response: agree (31%) or strongly agree (61.9%). Healthcare providers support carrier screening programs and provide appropriate education, and adequate support and time constraints are addressed [24]. The majority of the participants agreed (59.5%) or strongly agreed (31%) to the fact that counseling should be well-emphasized to the proband and the family with the help of audio-visual aids and charts. It will help the family to make a well-informed decision. A study assessing the impact of educational videos on genetic counseling outcomes showed that both genetic counselors and families found the videos to be beneficial [25]. There was a mixed response, namely, strongly disagree (7.1%), disagree (9.5%), neutral (11.9%), agree (52.4%), and strongly agree (19%), for informing the patient to do appropriate genetic tests for risk determination of disease transmission. This fact is indeed influenced by the specialty, knowledge, and experience of the practicing physician.

Limitations of the study 

Knowledge and attitude could not be completely assessed in this study since routine genetic counseling has to be done in multiple sittings and at length. Moreover, other components of counseling, such as advice for appropriate genetic tests, referral to support groups, and follow-up strategies, are not assessed in the present study. Therefore, the depth of the practice of genetic counseling by clinicians should be further evaluated in detail as a continuation of this study.

## Conclusions

Rapid advances in molecular genetics have influenced the clinical management of the disease with genetic etiology. In the present era, clinicians in India have in-depth knowledge about the genetic basis of diseases. They do agree to the fact that the patient and the family should be counseled at length for a particular genetic disorder. Very little research exists on the preaching and practice of genetic counseling by clinicians. Disorders having underlying genetic causes can present at any age and can involve any system of the body. Therefore, it is important for primary physicians and specialists to diagnose a genetic disorder, offer genetic counseling, and develop the necessary expertise in the field.
